# Nested singletons in molecular trees: Utility of adding morphological and geographical data from digitized herbarium specimens to test taxon concepts at species level in the case of *Casearia* (Salicaceae)

**DOI:** 10.1002/ece3.9736

**Published:** 2023-01-17

**Authors:** Astrid de Mestier, Robert Lücking, Jorge Gutierrez, Grischa Brokamp, Marcela Celis, Thomas Borsch

**Affiliations:** ^1^ Botanischer Garten Freie Universität Berlin Berlin Germany; ^2^ Institut für Biologie – Systematische Botanik und Pflanzengeographie Freie Universität Berlin Berlin Germany; ^3^ Jardín Botánico Nacional Calabazar Universidad de La Habana Boyeros Cuba; ^4^ Fachbereich Wald und Umwelt Hochschule für Nachhaltige Entwicklung Eberswalde Eberswalde Germany; ^5^ Departamento de Química y Biología Universidad del Norte Barranquilla Colombia

**Keywords:** Ecological niche equivalency test, integrative taxonomy, molecular phylogeny, morphometry, PCA, species delimitation

## Abstract

Using the genus *Casearia*, we assessed the status of nested singletons: individual specimens corresponding to accepted species but in molecular trees appearing nested within clades of closely related species. Normally, such cases would be left undecided, while on the other hand, timely taxonomic decisions are required. We argue that morphological, chorological, and ecological data can be informative to illuminate patterns of speciation. Their use can provide a first step in testing taxon concepts at species level. We focused on five cases of nested singletons in trees of the genus *Casearia*. We employed PCA and cluster analysis to assess phenotypic differentiation. Using geocoordinates, we calculated niche space differentiation based on 19 bioclim variables, by means of PCA and niche equivalency and similarity tests and generated dot maps. We found that the singletons were morphologically distinctive in two of the five cases (*Casearia selloana* and *C. manausensis*), relatively distinctive in two other cases (*C. zizyphoides* and *C. mariquitensis*), and partially overlapping in the last case (*C. grandiflora*). For two cases (*C. mariquitensis* and *C. selloana*), ecological niche space was broadly overlapping, in two cases it was found broadly nested (*C. grandiflora* and *C. zizyphoides*), and in one case narrowly nested (*C. manausensis*), but in no case niche differentiation was observed. Niche overlap, similarity and equivalency showed corresponding patterns. Given these data, one would interpret *C. selloana* and *C. manausensis* as presumably well‐distinguished taxa, their narrow distribution ranges suggesting recently emerging lineages. The other three cases are not clearcut. Morphological data would suggest particularly *C. grandiflora* conspecific with *C. arborea*, but differences in the distribution are intriguing. Our approach would reject the notion of potential synonymy based on nested phylogenetic placement for at least two of the five cases. The other case also shows no complete lack of differentiation which would support synonymy.

## Graphical abstract

Using the genus *Casearia*, we assessed the taxonomical status of nested singletons: individual specimens corresponding to accepted species but in molecular trees appearing nested within clades of closely related species.

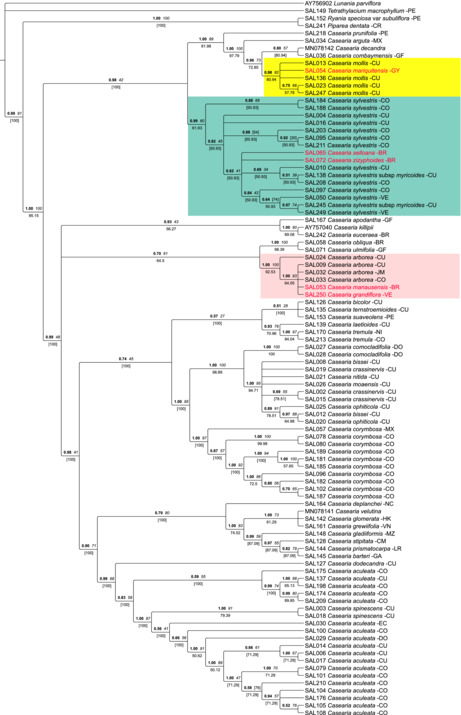

## 1 INTRODUCTION

Delimiting species is a challenging task, with respect to disparate species concepts that range from morpho‐species to different approaches reflecting complex speciation mechanisms in plants, but also in practical terms, regarding sufficient sampling of characters and individuals to make reliable assessments (Comes, 2004; Naciri & Linder, 2015). Historically, alpha‐taxonomy in plant species has been done based on the phenotype, using putatively diagnostic morphological characters shared among individuals presumed to belong to a thus‐defined species, denoting a morpho‐species concept (Stuessy, 2009). This approach has been implemented by the taxonomic community since the raise of formal species descriptions named with binomials in the mid of the 18th century (Linnaeus, 1753). Following the advent of phylogenetic systematics (Hennig, 1950, 1966) which provided a method to infer ancestor–descendant relationships and thus to reconstruct the history of species diversifications (Hillis, 1987). DNA sequences information is now routinely used in studies on evolution, systematics, and biogeography, yielding large numbers of molecular trees. The taxonomy of plants, as well as that of other organisms, is now in a transitionary phase from alpha taxonomy that recognized species based on the comparison of morphological characters, to the application of evolutionary methods in order to first infer distinct biological entities, which subsequently can be formally classified and named. However, such an evolutionary approach has only been thoroughly applied to a limited number of taxa. Current classification systems at the genus and even more so at the species level, which exhibit a mixture of taxa still defined on morpho‐species concepts, whereas others have been evaluated employing evolutionary methods (Borsch et al., 2015). In this ongoing process of taxonomic knowledge generation, species limits and the corresponding taxon concepts at species level are tested and eventually adjusted.

DNA sequencing has in many cases challenged traditional taxon concepts at species level, either unveiling that molecular phylogenies do not agree with the morphology‐based classification as evident by terminal clades with samples identified with one species name contain also samples identified with other currently accepted names. The use of DNA can unravel instances of cryptic speciation, when entities that share similar phenotypes while the respective individuals are found to be phylogenetically distinct (Simpson, 1951; Fišer et al., 2018) but also indicate that taxa currently accepted as different by morphology‐based treatments may in fact not represent different species. One of the major challenges is to utilize information from phylogenetic trees to revise taxonomic treatments to overcome the phylogeny to classification gap (Mayo et al., 2008; Hinchliff et al., 2015). Users of biodiversity information need to rely on biologically meaningful species classification in a most timely manner (Vogel Ely et al., 2017; Supple & Shapiro, 2018; Stanton et al., 2019), but at the same time cannot wait until all species limits will have been eventually clarified on the basis of densely sampled phylogenomic data sets. At the same time, a wealth of specimen information is currently becoming available in a digital form from herbaria worldwide (Thiers et al., 2016; Le Bras et al., 2017; De Smedt et al., 2018; Klazenga, 2018; Seregin, 2018) so it becomes feasible to explore morphological and geographical evidence for putative taxa using many specimens.

The state of knowledge for most genera is that molecular phylogenetic trees represent a great proportion of currently accepted species. Serving the goal of delivering first overall hypotheses of species relationships (Mansion et al., 2012), they usually represent species by single or few individuals and find different levels of resolution and node support in different parts of the tree. A frequent case in such molecular phylogenetic trees is the nested placement of singletons (i.e., individual samples representing a currently accepted species) within clades composed by individuals of another species. The existence of paraphyletic species as a result of peri‐ or parapatric speciation also involving incomplete lineage sorting is now widely accepted (Crisp & Chandler, 1996; Hörandl & Stuessy, 2010; Carnicero et al., 2019; Kato et al., 2019). Nested singletons could therefore represent biologically distinct entities, deserving recognition at species level, or just represent an own haplo‐ or genotypes, thus exhibiting infraspecific genetic diversity of the so far better represented species in the molecular phylogeny. The occurrence of singletons usually corresponds to the rarity of the corresponding taxon and the difficulty to access suitable material. And often they belong to recently diverged shallow clades that show a lack of phylogenetic differentiation relative to the apparent phenotypic or ecological differentiation (Lexer & Widmer, 2008; Ravinet et al., 2017). Species may vary considerably in their infraspecific phylogenetic structure when multiple individuals from different populations throughout their geographic range are included (Borsch et al., 2018). Despite their frequent occurrence (see for example trees published by Bengtson et al., 2021; Frost et al., 2021; Lu‐Irving et al., 2021; Majure et al., 2021, García‐Moro et al., 2022), cases of nested singletons are almost never discussed.

In addition to evidence from molecular trees, the morphological, ecological, and/or chorological differentiation is relevant to get further insights if putative taxa represented by single sequences are biologically distinct entities. Such an integrative taxonomy approach is being increasingly used (Dayrat, 2005; Will et al., 2005; Padial et al., 2010; Schlick‐Steiner et al., 2010; Pante et al., 2015). A fundamental principle of integrative taxonomy is to generate specimen‐based character data (Kilian et al., 2015) that can allow precise testing of the placement of individuals (i.e. individual samples) in an evolutionary context when subjected to different inference methods. By using the geographical occurrence points of the respective specimens, ecological parameters can also be assessed and integrated in models of distribution and species delimitation.

Here, we address a different situation that occurs during taxonomic work that needs to deliver the best possible judgment of species limits during treatments of genera or even families at global or regional levels, in the course of which a comprehensive molecular analysis is not realistic for time, capacity, material availability and resource reasons. Our use of an integrative taxonomic approach is, therefore, based on the assumption that morphological, geographical, and ecological data still show a pattern related to the evolutionary history of the putative species under study (Thompson et al., 2005). The advantage of these data is that they can be obtained for a large and representative number of specimens, now facilitated by herbarium digitization. Those individuals represented by sequences in the molecular tree and investigated at the same time for the nonmolecular data constitute a link between the available phylogenetic hypotheses and entities discovered by analyzing the nonmolecular data (PCA or clustering algorithms, spatial and ecological models). We analyze currently accepted taxa revealed as nested singletons in a recent molecular phylogenetic analysis of the genus *Casearia* Jacq. (Mestier et al., 2022) within the presumably widespread and common species *C. arborea* (Rich.) Urb., *C. mollis* Kunth, and *C. sylvestris* Sw.

*Casearia* is a pantropical genus that comprises around 220 species of shrubs or trees, half of which are found in the Neotropics (Sleumer, 1980). It is the largest genus within a broadly defined Salicaceae, including the tribe Samydeae, which is sometimes classified at the rank of family (Alford, 2005). *Casearia* has alternate, serrate leaves that present pellucid dots and/or lines and flowers in axillary and usually fasciculate inflorescences. The flowers are apetalous with five sepals and they present staminodes, alternating with the stamen or sometimes inserted outside of the row of stamen (Warburg, 1895; Sleumer, 1980). Most species are widely distributed and found across various habitats in the Neotropics, including Amazonian rainforests, Brazilian cerrados (Sleumer, 1980; Gutiérrez, 2000; Marquete & Mansano, 2012), dry forest (DRYFLOR et al., 2016), or savannas (Devecchi et al., 2020), whereas others are considered range restricted (Breteler, 2008) or endemic (Marquete & Mansano, 2010; Applequist & Gates, 2020). About 30 species occur in the Caribbean (Sleumer, 1980; Correll & Correll, 1982; Howard, 1989; Liogier, 1994; Gutiérrez, 2000), which have evolved as a result of multiple migrations of ancestors to the islands since the late Miocene (Mestier et al., 2022). Sleumer (1980) provided the so far most complete revision of the genus in the Neotropics, but some species remain unclear.

The specific objectives of this study are to evaluate the degree of phenotypic differentiation, differentiated distribution, and ecological niche differentiation, for five currently accepted species‐level taxa of *Casearia* appearing as part of terminal clades composed of individuals of *C. arborea* (Rich.) Urb., *C. mollis* Kunth, and *C. sylvestris* Sw. in comparison to their widespread relatives. We included all available herbarium specimens that could be reliably assigned to the respective, currently accepted taxa. Based on PCA and clustering analysis for the morphological data and distribution and niche space analyses our goal is to explore in how far such nonmolecular evidence can help to delimit species and thus can be used to support the circumscription of taxon concepts at species level. Moreover, our aim is to discuss our findings considering the current implementation integrative approaches in flowering plant taxonomy.

## 2 MATERIAL AND METHODS

### 2.1 Taxon sampling and phylogenetic reconstruction

Our phylogenetic reference tree was based on the combined *rps4‐trnLF*, *trnK‐matK, petD,* and *rpl16* and the nuclear data set of Mestier et al. (2022). For the present investigation, we added 11 newly generated sequences of available relevant samples (voucher information in Appendix S1). Laboratory protocols were followed as in Mestier et al. (2022). We finally decided not to add further sequences downloaded from NCBI although the potential of this source was evaluated. However, vouchers were either not available online to allow for checking the identification or the respective specimens were not sequenced for the majority of the genomic regions used here for tree inference.

The alignment by Mestier et al. (2022) was used to incorporate the further sequences (Appendix S2 for alignments) implementing a motif‐alignment approach (see Löhne & Borsch 2005) in PhyDE (Müller et al., 2010). Short regions of uncertain homology (hotspots) were excluded from the analyses, and gaps were coded using the simple indel coding method (Simmons & Ochoterena, 2000) as implemented in SeqState version 1.4.1 (Appendix S3 for matrices used in tree inference).

We used MrBayes v.3.2.7.a (Ronquist et al., 2011) for Bayesian inference (BI). The optimal nucleotide substitution models were chosen using jModelTest v.2.1.7 (Darriba et al., 2012) under the Akaike information criterion (AIC). The best‐fit model for each partition can be found in Table 1. For the indels, the F81 model was used, as suggested by Ronquist et al. (2011). Four runs were performed with four chains and 40 million generations. Convergence of the runs was verified using the average standard deviation of split frequencies and post burn‐in effective sampling size (ESS). As a burn‐in, the first 10% of the trees were discarded, and the remaining trees were used to construct a 50% majority‐rule consensus tree. Maximum likelihood (ML) was implemented in RAxML v. 8.2.12. Rapid bootstrap support (BS) was estimated based on the majority‐rule consensus tree from 1000 pseudo‐replicates with 200 searches. The models *general time‐reversible (GTR) + τ* and *binary (BIN) + τ*, respectively, were used in nucleotide and indel partitioning. All those analyses were realized through the CIPRES portal (Miller et al., 2011). The ML phylogram was illustrated in FigTree v1.4.4 (Rambaut, 2010). We performed parsimony analysis (P) in PAUP* v.4.0b10 (Swofford, 2008) using the commands obtained from the parsimony ratchet (Nixon, 1999) as implemented in PRAP (Müller, 2004). PRAP generated files including all characters with equal weight and the gaps were treated as missing characters. Ratchet setting included 200 iterations, unweighting 25% of the positions randomly (weight = 2) and 100 additional cycles. Jackknife support (JK) was obtained through a single heuristic search in PAUP within each of 10,000 JK pseudo‐replicates, tree bisection‐reconnection branch swapping, and 36.79% of characters being deleted in each replicate. All trees were processed using TreeGraph 2 (Stöver & Müller, 2010), and node support values of all inference methods were depicted on the Bayesian majority rule topology.

**TABLE 1:** Summary of character statistics, evolutionary models, and trees statistics for each dataset under maximum parsimony, maximum likelihood, and Bayesian inference

	rps4‐trnLF	trnK‐matK	petD	rpl16	Combined	Nuclear
Number of taxa	96	96	96	96	96	66
Aligned length (bp)	2133	3137	1334	1128	7732	900
Constant characters (proportion)	0.82	0.79	0.76	0.71	0.79	0.52
Parsimony‐informative characters	178	345	178	164	795	239
Consistency index (CI)	0.802	0.78	0.728	0.75	0.662	0.572
Retention index (RI)	0.903	0.877	0.864	0.87	0.793	0.78
Tree length	494	970	515	488	2709	968
Partitions	Spacer rps ‐ trnTexon	trnK intron	petBexon ‐spacer	rpl16		
	Spacer trnT‐trnL ‐ trnL exon	matK	petD intron			
	trnL intron ‐ spacer trnL trnF ‐ trnF	trnK intron 2‐trnK‐exon 2‐spacer ‐ psbA				

### 2.2 Target taxa

The following cases of nested singletons were selected for study. *C. grandiflora* Cambess and *C. manausensis* Sleumer nested within *C. arborea* (Rich.) Urb.; *C. selloana* Eichler and *C. zizyphoides* Kunth nested within *C. sylvestris* Sw., and *C. mariquitensis* Kunth being part of the *C. mollis* Kunth clade. Our sampling of these deviant taxa has been limited due to the availability of material, and thus they are so‐called “singletons.” Mestier et al. (2022) also retrieved *C. spinescens* nested within *C. aculeata*, however, given the incongruence between plastid and nuclear trees, where *C. spinescens* is retrieved as sister to *C. aculeata*, we chose to not further analyze it here.

### 2.3 Locality data

Using a set of specimens corresponding to the above taxa following the taxon concept at species level sensu Sleumer (1980), we compiled occurrence data from different herbaria (B, COL, FMB, HEUS, HUA, JAUM, JBGP, MEDEL, MEXU, NY, UNO, UTMC) Flora do Brasil (https://floradobrasil.jbrj.gov.br/) and GBIF (https://www.gbif.org/). Recently, the development of herbarium digitalization (James et al., 2018; Rønsted et al., 2020) facilitated access to distribution but also morphological data available through GBIF (Robertson et al., 2014). For GBIF data, we filtered for specimen‐based occurrences only. We only considered specimen records identified by specialists for *Casearia* and allies, or those with digital voucher images for which we could verify the identification. We manually verified that coordinates matched with corresponding localities. Missing coordinates were added when locality data were precise enough to allow for reliable georeferentiation. For Colombian samples, we used centroid coordinates of either municipalities, veredas, natural parks, or reserves, following the administrative divisions of Colombia (DANE, 2017). For the remaining samples, we used Google Earth (GoogleInc., 2020). We then deleted duplicate specimen, filtering the data by coordinates and localities using R v4.0.3 (RCoreTeam, 2013).

### 2.4 Morphological analyses

Based on directly inspected vouchers or digital specimens from the herbaria of B, HAJB, K, NY, UNO, P, and Jstor Global Plants, a total of 200 specimens were analyzed morphologically, 60 of *C. arborea,* 36 of *C. grandiflora,* 13 of *C. manausensis,* 15 of *C. mariquitensis,* 22 of *C. mollis,* 10 of *C. selloana,* 34 of *C. sylvestris,* and 10 of *C. zizyphoides*. (Table 2). For all specimens, we examined the length and the width of the leaf, as well as the length of the petiole. Further characters were specifically studied for each pair of nested vs. the corresponding paraphyletic taxon, indicated as being diagnostic in taxonomic treatments (Sleumer, 1980; Olson et al., 1999; Nepomuceno & Alves, 2020). Quantitative measurements were performed using the digital image analyses software ImageJ 1.53a (Schneider et al., 2012). We computed descriptive statistics for all quantitative variables (mean, standard deviation). For categorical variables, we used the “fastdummie*s”* package (Kaplan, 2020) in R v.4.0.3 (RCoreTeam, 2013), which transforms the variables into binary variables, recoding states as presence/absence variables. We employed principal component analysis (PCA) and cluster analyses using the Ward.D2 method with the NbClust package (Charrad et al., 2014) to analyze the character matrices for nested versus corresponding paraphyletic taxon pairs in multivariate fashion. All information regarding the specimens and the respective measurements can be found in Table S1.

**TABLE 2:** Morphological characters for each species analyzed

	C. arborea	C. grandiflora	C. manausensis	C. mariquitensis	C. mollis	C. selloana	C. sylvestris	C. Zizyphoides
Leaves margins	Serrulate	Crenulate to serrate	Subserrate to crenate	Serrate	Serrate	Entire to serrate	Subentire to serrate	Subentire
Leaves pilosity	Glabrous	Tomentellous beneath	Hirsutous	Glabrous	Tomentellous beneath	Glabrous	Glabrous	Glabrous
Leaves color when dry	Brown	Brown	Light brown	Brown	‐	‐	‐	‐
Discolorous	Presence	Presence	Absence	Absence	Absence	Absence	Absence	Absence
Inflorescence	Pedicellate	Sessile	Subsessile	Pedicellate	Pedicellate	Pedicellate	Pedicellate	Pedicellate
Flower number	Up to 15	Up to 15	6–10	10–15	More than 15	Many	Many	10–15
Leaves punctuation	Presence	Presence	Presence	Presence	Presence	Impunctate with age	Densely punctate and lineate	Presence
Mucron	Absence	Absence	Absence	Absence	Absence	Absence	Absence	Presence
Tip of the leave	Acuminate, acute	Acuminate	Acute	Subcaudate to acuminate	Acuminate	Acuminate	Acuminate to acute	
Style	Entire	Entire		Entire	Entire	3‐parted	3‐parted	Entire
Source	Sleumer, (1980); protologue, type	Sleumer, (1980); protologue, type	Sleumer, (1980); protologue, type	Sleumer, (1980); Olson, 1999	Sleumer, (1980); Olson, 1999	Sleumer, (1980)	Sleumer, (1980)	Sleumer, (1980)

*Note*: Flowering characters are presented for general information but are not used in the analyses. Discolorous: Superior side of the limb darker than the inferior side (presence/absence), leaves pilosity: Presence (or absence) and type of pubescens on the limb, tip of the leaves: Tip shape, style: Entire or parted.

### 2.5 Environmental niche space analysis

To test divergence in environmental niche space between nested vs. including taxon, we obtained 19 climatic layers from WorldClim at 1 km^2^ resolution (http:/www.worldclim.org/bioclim). A shape layer was generated by cropping the grid data to the area of the Neotropics using R v.4.0.3 (RCoreTeam, 2013). In order to reduce complexity and avoid overparametrization, we carried out a collinearity test, using the Pearson correlation coefficient from the “remove Collinearity” function of the “VirtualSpecies” package(Leroy et al., 2016), with a cutoff value that we set at 0.75. We selected one for each group of correlated environmental variables, usually the variable representing the annual trend (mean). This reduced the data set to nine climatic layers (Table S2).

We retrieved data for a total of 931 occurrences (information regarding the specimen used can be found in Table S3). From these, 219 belonged to *C. arborea,* 168 to *C. grandiflora,* 12 to *C. manausensis,* 105 to *C. mariquitensis,* 33 to *C. mollis,* 39 to *C. selloana*, 324 to *C. sylvestris,* and 33 to *C. zizyphoide*.

Based on the georeferenced locality data for specimens representing each taxon, we realized PCA analyses to visualize potential differences in the ecology between pairs of taxa. To assess niche equivalency and similarity, we used the “Ecospat” package (Di Cola et al., 2017). First, we computed the Schoener's D statistic, to quantify niche overlap between pairs of species, ranging between 0 for no overlap in environmental space and 1 for identical environmental space. Given that in the case of allopatric species, geographical differences might lead to differences in the environmental conditions available, we conducted a niche similarity test, which used the model of one species to predict the occurrence of the second species (Warren et al., 2010). Information regarding the specimens used for the analyses can be found in Table S3.

### 2.6 Distribution maps

We generated distribution maps with the geographic information software QGIS 3.10 (QGIS association, 2020), using the locality data of specimens with verified identification and locality data from local flora to cover the entire range of a species distribution, even when no specimens were available with reliable coordinates (Table S3), These were drawn by nested vs. including taxon pairs in order to observe potential geographic differentiation.

## 3 RESULTS

### 3.1 Molecular data sets

The concatenated multiple sequence alignment of the four plastid genomic regions had 7731 positions, of which *rps4‐trnT‐L‐F* contributed 2133 *trnK‐matK* 3138, *petD* 1333, and *rpl16* 1127 (Appendix S2, Table 1). The final matrix consisted of 8024 positions (21% variable, 10% parsimony informative including 293 indels after exclusion of hotspots (Appendix S3; Table 1). The alignment of the ITS region had 762 positions (Appendix S4, Table 1). The final matrix contained 900 positions (48%variable, 26.5% informative) including 138 indels, after exclusion of hotspots (Appendix S5, Table 1).

### 3.2 Phylogenetic relationships of *Casearia* and positions of nested singletons

**FIGURE 1:**
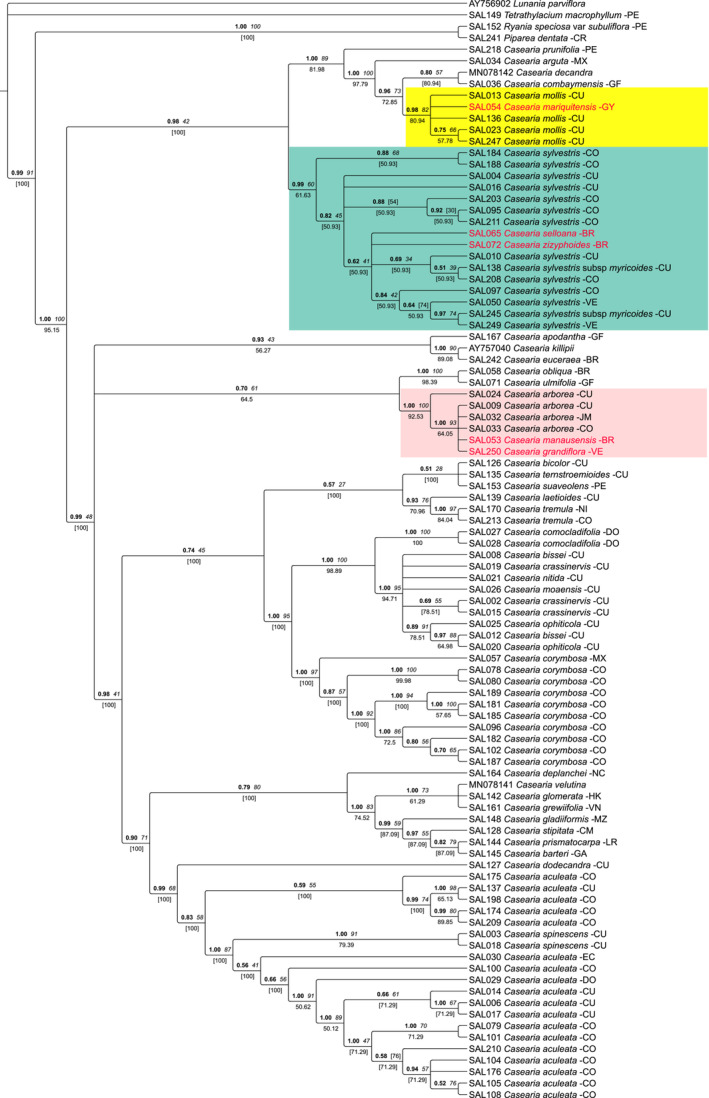
Bayesian 50% majority‐rule consensus tree of *Casearia* based on four plastid markers (*rps4/trnL‐F, trnK/matK, rpl16, petD*). Values above the node indicate posterior probability (PP, bold) and bootstrap support (BS, italics), and jackknife (JF) support is indicated below the node. In square brackets are the values with conflicting topologies between Bayesian analysis and parsimony. The tip of the node indicate the DNA number followed by the name of the species and the code of the country where the individual was collected.

The plastid topology is shown in Figure 1 and provides a well‐resolved phylogenetic framework for the monophyletic genus *Casearia* in line with Mestier et al. (2022). All three terminal clades (highlighted with colors) of widespread *Casearia* species with nested singletons are well supported. *Casearia mariquitensis* from Guyana was found as part of the *C. mollis* clade (BI‐PP: 0.98, BS: 82, JK: 80.94). The single specimens of *Casearia selloana* from Brazil and *C. zizyphoides* from Venezuela were retrieved in a core polytomy within the *C. sylvestris* clade (crown group BI‐PP: 0.99, BS: 60, JK: 61.63) including samples from the Caribbean and South America. Moreover, we retrieved one sample of *C. grandiflora* from Venezuela and one sample of *C. manausensis* from Brazil within the highly supported *C. arborea* clade (BS: 1, BS: 100, JK: 92.53) otherwise including samples from the Caribbean and South America (Figure 1). Apart from that, our tree provides evidence for the monophyly of the widespread species *C. aculeata* and *C. corymbosa*, each of them showing considerable infraspecific phylogenetic structure.

**FIGURE 2:**
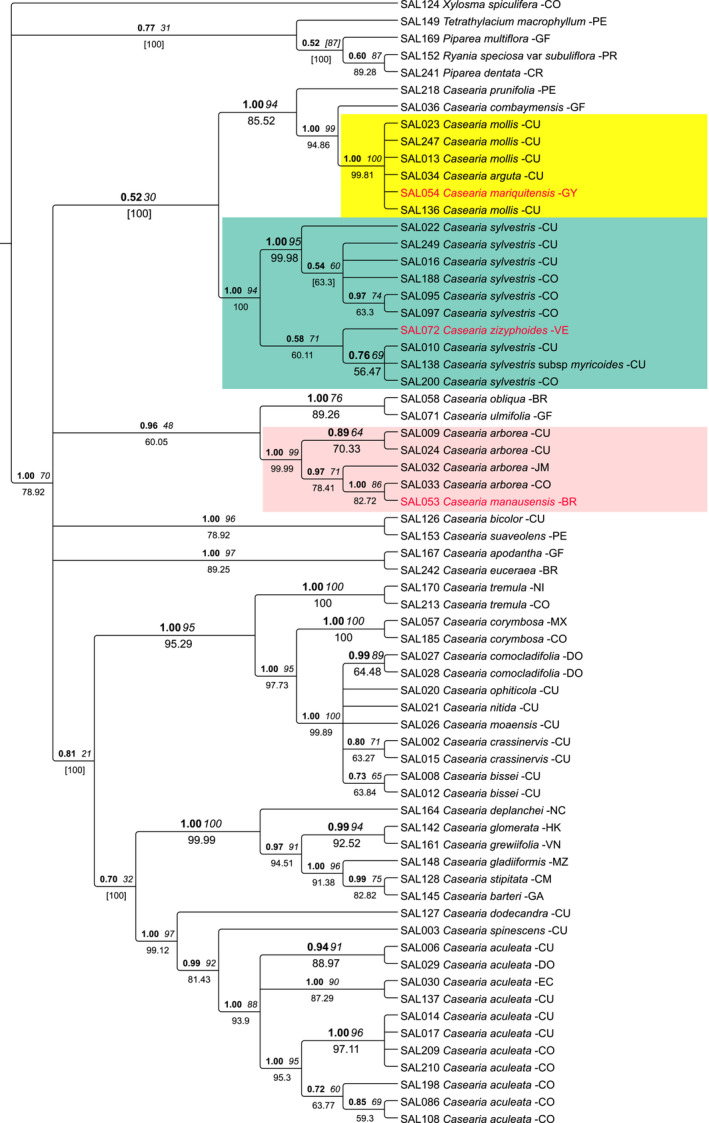
Bayesian 50% majority‐rule consensus tree of *Casearia* based on the nuclear marker ITS. Values above the node indicate posterior probability (PP, bold) and bootstrap support (BS, italics), and jackknife (JF) support is indicated below the node. In square brackets are the values with conflicting topologies between Bayesian analysis and parsimony. The tip of the node indicate the DNA number followed by the name of the species and the code of the country where the individual was collected.

The trees based on nuclear ITS were congruent with the plastid trees (Figure 2). *Casearia mariquitensis* was retrieved with good support within the *C. mollis* clade (BI‐PP:1, BS: 99, JK: 99.83), as well as *C. manausensis* within the *C. arborea* clade (BI‐PP:1, BS: 86, JK: 82.72) and *C. zizyphoides* within the *C. sylvestris c*lade (BI‐PP: 1, BS: 94, JK: 99.98). Unfortunately, we were unable to amplify the nuclear marker for the remaining two nested singletons.

### 3.3 Morphological analyses

**FIGURE 3:**
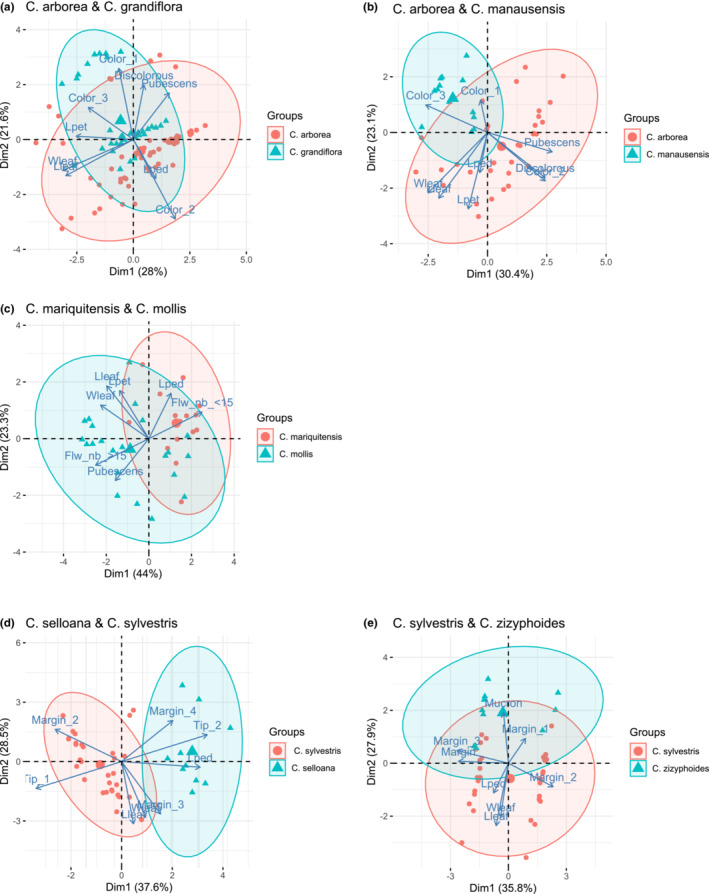
PCA plot based on selected morphological characters for pairs of species: (a) *C. arborea* and *C. grandiflora,* (b) *C. arborea* and *C. manausensis,* (c) *C. mariquitensis* and *C. mollis*, (d) *C. selloana* and *C. sylvestris*, (e) *C. sylvestris* and *C. zizyphoides*. Points represent individuals, arrows individual parameters. Lleaf: Limb length, Wleaf: Limb width, Lpet: Petiole length, Lped: Pedicel length (or presence/absence for a), margin 1/2/3/4: Margins crenate/subentire/serrate/entire, color 1/2/3: Leaf color when dry, green/brown/light brown, discholorous: Superior side of the limb darker than the inferior side (presence/absence), pubescens: Presence of pubescens on the limb (presence/absence), Flw nb <15: Up to 15 flowers (presence/absence), Flw >15: Between 15 and 30 flowers (presence/absence), tip 1/2: Tip shape, acuminate/acute, Mucron (presence/absence)

**FIGURE 4:**
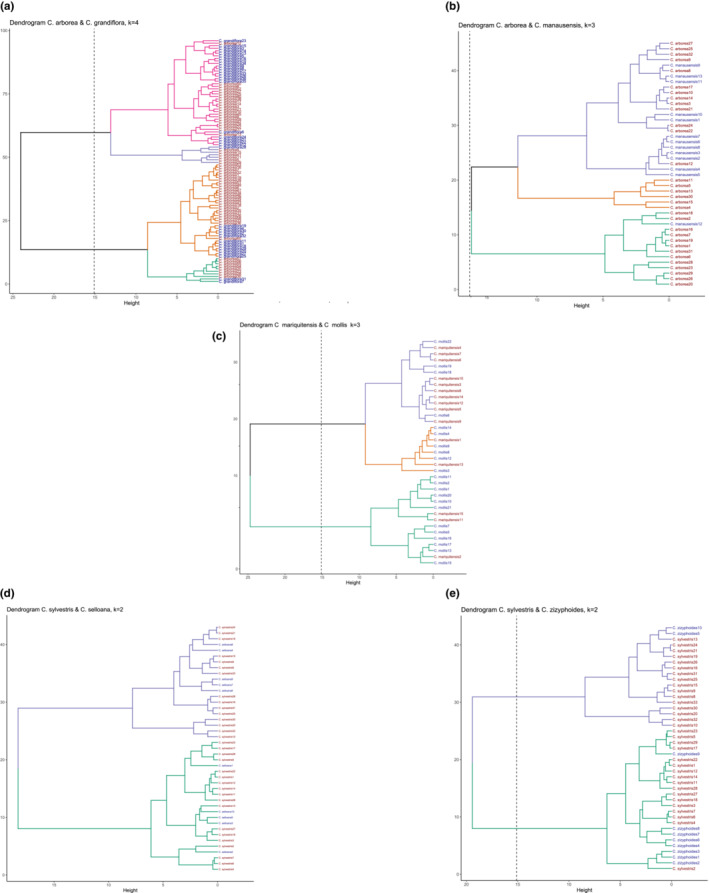
Dendrograms plots for pair of species (a) *C. arborea* and *C. grandiflora*, (b) *C. arborea* and *C. manausensis*, (c) *C. mariquitensis* and *C. mollis*, (d) *C. selloana* and *C. sylvestris*, (e) *C. sylvestris* and *C. zizyphoides* on morphological characters

The PCA analysis of *C. grandiflora* versus *C. arborea* showed a morphological overlap, but a strong tendency of differentiation along the two perpendicular axes (Figure 3a), whereas cluster analysis revealed four distinct groups that did not coincide with the two species (Figure 4a). In this case, distribution of individuals between the main clusters was rather homogeneous. The morphological overlap between *C. manausensis* versus *C. arborea* was less pronounced than in the previous case (Figure 3b) and cluster analysis indicated a nested structure, with most specimens of *C. manausensis* placed in one of the groups but mixed with specimens of *C. arborea* (Figure 4b). For *C. mariquitensis* versus *C. mollis,* PCA showed some morphological overlap (Figure 3c) and the cluster analysis supported no distinction. Cluster 1 contained 71% *C. mariquitensis* and 29% *C. mollis,* whereas cluster 2 consisted of 75% *C. mollis* and 25% *C. mariquitensis* and cluster 3 of 80% *C. mollis* and 20% *C. mariquitensis* (Figure 4c). A different pattern was found for *C. selloana* versus *C. sylvestris*, with little overlap in the PCA analysis (Figure 3d), although this distinction was less obvious in the cluster analysis (Figure 4d). *Casearia selloana* thereby seemed to present longer petioles with entire margins and an acute tip. For *C. zizyphoides* versus *C. sylvestris,* PCA also showed limited overlap (Figure 3e), whereas in the cluster analysis, we retrieved two clusters that did not correspond to the two taxa (Figure 4e).

### 3.4 Environmental niche space analysis

**FIGURE 5:**
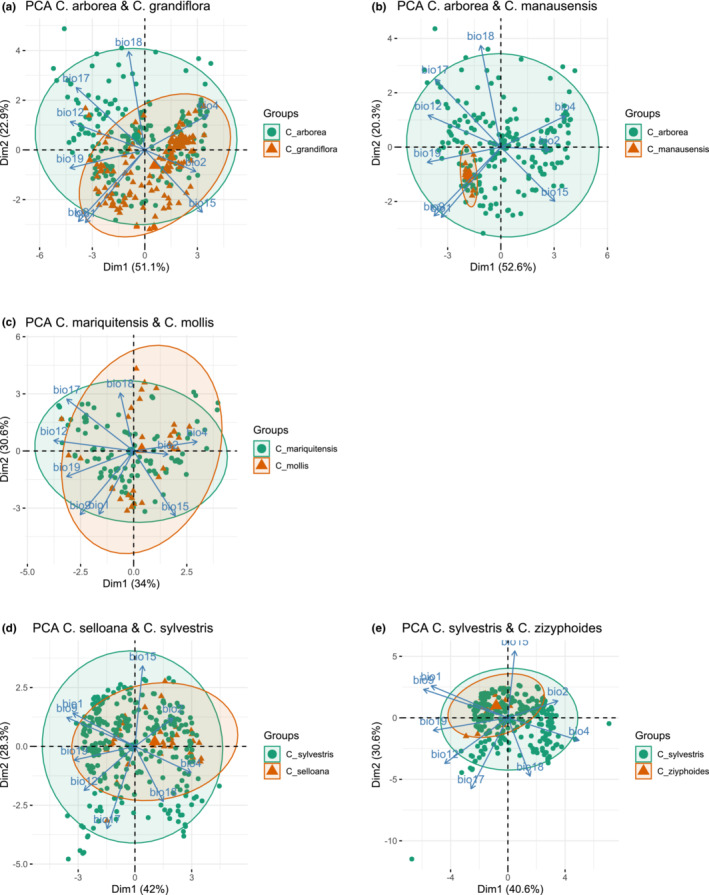
PCA plot based on selected ecological variables from WorldClim for pairs of species: (a) *C. arborea* and *C. grandiflora,* (b) *C. arborea* and *C. manausensis*, (c) *C. mariquitensis* and *C. mollis,* (d) *C. selloana* and *C. sylvestris,* (e) *C. sylvestris* and *C. zizyphoides*. Points represent individuals, arrows individual parameters

The results for *C. grandiflora* and *C. arborea* showed considerable ecological overlap (Figure 5a). In the case of *C. manausensis* versus *C. arborea,* the PCA analysis showed a pattern with individuals from *C. manausensis* being nested within *C. arborea*, i.e. pointing to a much narrower ecological niche of *C. manausensis* (Figure 5b). For *C. mariquitensis* and *C. mollis*, we also retrieved a high ecological overlap in the PCA analysis (Figure 5c). *Casearia selloana* and *C. sylvestris* also presented no discernible ecological differentiation (Figure 5d), and the same pattern was found for *C. sylvestris* and *C. zizyphoides* (Figure 5e).

Niche similarity tests were significant for all cases of paired species; but one, as *C. arborea* and *C. grandiflora* showed no niche similarity, nor equivalence. For the other four pair, the niche similarity was always higher than expected by chance (Table 3). Highest niche overlap was 0.70 for *C. mariquitensis* versus *C. mollis* with a significant level of niche equivalence (Table 3). For the remaining pairs, niche equivalence was not significant and niche overlap was lower, ranging from 0.43 in *C. grandiflora* versus *C. arborea* to as low as 0.02 in *C. manausensis* versus *C. arborea*, with *C. selloana* versus *C. sylvestris* (0.26) and *C. zizyphoides* versus *C. sylvestris* (0.20) inbetween (Table 3).

**TABLE 3:** Results of the ecological niche analysis

Species		Niche overlap (D)	Niche similarity		Niche equivalency
a	b		Greater	Lower	
*C. arborea*	*C. grandiflora*	0.44	*p* = .06	*p* = .92	ns
*C. arborea*	*C. manausensis*	0.04	***p* = .003**	*p* = 1	ns
*C. mariquitensis*	*C. mollis*	0.7	***p* = .004**	*p* = 1	***p* = .01**
*C. sylvestris*	*C. selloana*	0.3	***p* = .001**	*p* = 1	ns
*C. sylvestris*	*C. zizyphoides*	0.47	***p* = .001**	*p* = 1	ns

*Note*: Values in bold indicate a significant result (*p* < .05).

Abbreviation: ns, Nonsignificant.

### 3.5 Distribution

**FIGURE 6:**
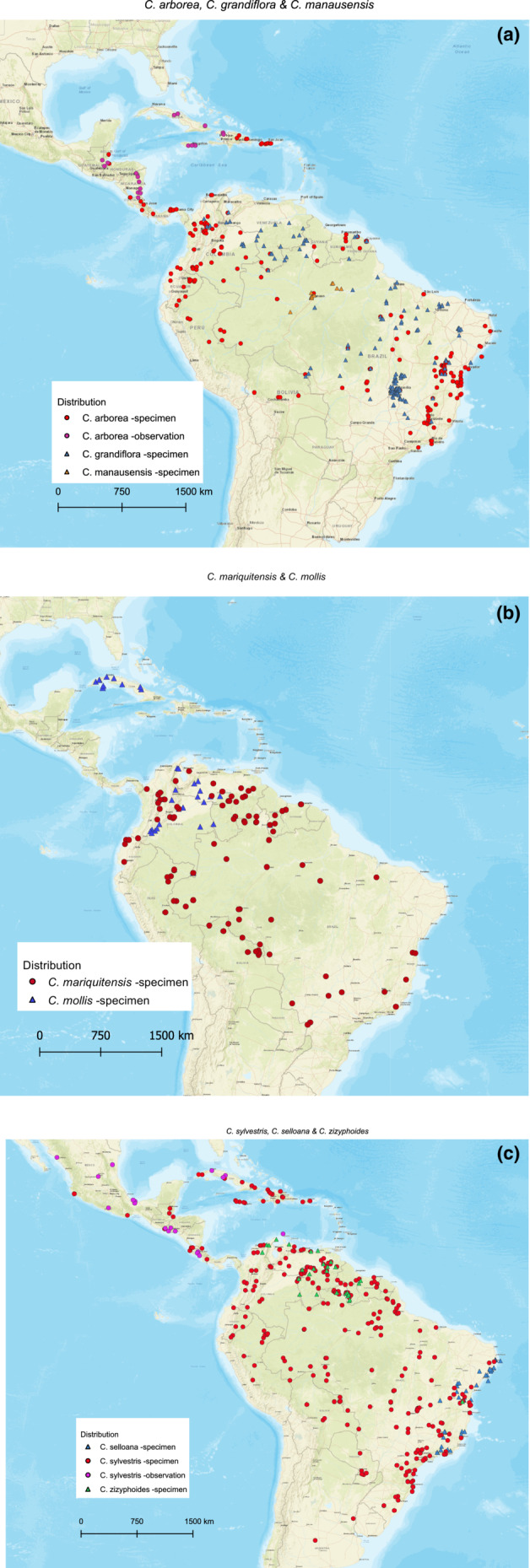
Distribution maps: (a) *C. arborea*, *C. grandiflora* and *C. manausensis*, (b) *C. mariquitensis* and *C. mollis*, (c) *C. selloana*, *C. sylvestris* and *C. zizyphoides*

*Casearia arborea* and *C. grandiflora* are both widely distributed. Whereas *C. arborea* is concentrated in the mountainous regions of the Northern Andes, Central America, the Caribbean, and the Brazilian Atlantic Forest, *C. grandiflora* is mostly found in the broader Amazon region in wet and dry forests (Figure 6a). *Casearia manausensis* has a narrow distribution within the broad range of *C. arborea*, reported only from a small area in the central Amazon, around Manaus (Figure 6a). *Casearia mariquitensis* and *C. mollis* are broadly overlapping in northern South America, but with *C. mollis* also present in Cuba, whereas *C. mariquitensis* extents further south in South America (Figure 6b). *Casearia sylvestris* is the most broadly distributed species in this study, being found across the entire Neotropics; in contrast, *C. selloana* is restricted to the northeastern Brazil, and *C. zizyphoides* is only found in northern South America (Figure 6c).

## 4 DISCUSSION

### 4.1 An integrative approach for species delimitation in the case of *Casearia*

Proper species delimitation is crucial not only for accurate biodiversity assessments and biodiversity monitoring but also for downstream studies, such as ecology and conservation (Agapow et al., 2004; Rojas‐Soto et al., 2010; Ruiz‐Sanchez & Londoño, 2017; Sheridan & Stuart, 2018). Mostly with respect to insufficiently resolved molecular trees or sampling gaps in molecular data sets, Edwards and Knowles (2014) and Mayo (2022) argue that an integrative taxonomy approach including additional kinds of data can help toward further assessing species limits. In our case of neotropical *Casearia* species, the molecular trees are inconclusive in depicting deviant species currently accepted based on morphology (e.g. Sleumer, 1980) as part of terminal clades of other species. While these species remain phylogenetically unresolved, we can reliably assume close relationships with the including species as annotated on the trees (Figures 1 and 2). Therefore, our resulting species‐level taxon pairs therefore provide a valid set up for the comparative analysis of morphological and ecological data, as well as for the comparison of their respective ranges, to test for differentiations not evident in the limited molecular data available. The nested position of the five study cases (*C. manausensis* and *C. grandiflora* within *C. arborea*; *C. mollis* within *C. mariquitensis*; *C. selloana* and *C. zizyphoides* within *C. sylvestris*), leaving the residual species paraphyletic, could be interpreted as a lack of resolution by the molecular markers applied so far. Resolving such situations with additional molecular data would be desirable, but for practical reasons is challenging due to the difficulties in targeted sampling. Therefore, in a taxonomic treatment based on the currently available data, a decision would have to be made in either situation as to recognize one or more taxa. Evidence from morphology, ecology, and geography could therefore be of fundamental importance to retain putatively distinct biological entities that warrant continued recognition as a distinct taxon.

In the case of *C. mariquitensis* versus *C. mollis*, there is some evidence for morphological differentiation between the two taxa, but there is a lack of niche differentiation and a broadly overlapping distribution, suggesting that *C. mollis* could be maintained as a species different from *C. mariquitensis* based on morphological features only. Individuals identified as *C. grandiflora* or *C. arborea* showed strong morphological differentiation, while exhibiting limited niche equivalency and niche similarity. In addition, although both appear widely distributed across the Neotropics, the first is more abundantly found in the Amazon and adjacent dry forests and the second more frequently in the Andes, the Atlantic forest, and Central America and the Caribbean. These patterns clearly support the continued acceptance of two separate taxa. *Casearia manausensis* was also strongly differentiated morphologically from *C. arborea* and was narrowly nested within the ecological niche space of the latter. Given that *C. manausensis* has only been recognized from a very small area within the range of *C. arborea*, it could thus represent an emerging lineage warranting taxonomic recognition. *Casearia selloana* presented a somewhat similar case, with strong morphological differentiation toward *C. sylvestris*, although there was only little ecological differentiation and the range of *C. selloana*, restricted to northeastern Brazil, was more broadly nested within that of the neotropical *C. sylvestris*. Lastly, *C. zizyphoides* was less well distinguished morphologically from *C. sylvestris* than *C. selloana*, but exhibited a more distinctly nested niche space and likewise a nested range, mostly restricted to the Guianas. Therefore, the various lines of evidence suggest that *C. selloana* and *C. zizyphoides* represent distinct taxa, separate from *C. sylvestris*, even if not obvious from the limited molecular data. Therefore, in all five tested cases, we argue to maintain the hitherto used classification rather than sinking the respective accepted names into synonymy, while highlighting the need for additional investigation (Scherz et al., 2017; Guenser et al., 2022).

### 4.2 Kinds of data used and their potential for taxonomic decision making

By integrating different data, a structured taxonomic decision‐making process can be supported. This requires evaluating the relative contributions of these different kinds of data. In this investigation, the molecular data mostly came from the recently presented phylogeny of *Casearia* (Mestier et al., 2022), with sequences here added for further individuals of *C. grandiflora*, *C. mollis*, and *C. sylvestris*. Despite of this, the now sequenced individuals still do not represent populations from throughout the ranges of the respective species nor do they fully cover the morphological variation encountered in the available herbarium specimens, which were obtained in decades of collecting in many countries. Therefore, the full assessment of molecular variation in a putative species throughout its assumed range was not possible due to a lack of adequate material in our *Casearia* exemplars. In light of possible infraspecific variation, more material would in fact be required. However, considering large neotropical ranges of most of the respective taxa, this would not have been feasible in any timely context that allowed to deliver the best possible treatment for syntheses like the World Flora Online (Borsch et al., 2020).

Our morphological and distributional data came in part from herbarium specimens serving as vouchers for our molecular analysis but were substantially extended by examining images and databased label metadata of specimens identified to the corresponding taxa by experts or used as vouchers in monographic treatments. Instead of working with physical specimens throughout, digitization of herbarium vouchers thereby greatly facilitated this approach, as we were able to include around 200 further specimens for morphometric analysis and around 900 georeferenced specimens for distribution analyses. The difference in number for the two kinds of data comes from the fact that morphological analyses required well‐preserved specimens with all critical features present and with digital images available, whereas for the distribution analysis, we also used specimens without images, if the identification had been made by a specialist and could be assumed to be trustworthy. A limitation to this approach was that some diagnostic characters could not be properly assessed on all digitized specimens, such as the density of pellucid dots which may be an additional diagnostic character for *C. selloana* (Sleumer, 1980). In this case, the morphological characters used in this investigation already showed a clear morphological distinction of *C. selloana* from *C. sylvestris*, so including this character would not have changed our conclusion.

We hypothesized that for a nested singleton to represent a separate species, it should present some phenotypic differentiation toward the taxon it is nested within. Bromham et al. (2002) pointed out that rates of phenotypic differentiation can be higher than substitution rates of the studied genomic markers. Phenotypic differentiation could therefore be an indicator of reproductive isolation and parapatric speciation. In addition, or alternatively, the ecological niche of the taxon in question should reveal differentiation, and finally a differential distribution range, in line with allo‐, peri‐, or parapatric speciation could be an indication of an emerging lineage even if not seen in the analyzed markers. In contrast, synonymy of previously distinguished taxa might be indicated if they present no morphological and ecological differentiation and if their distribution ranges are largely or entirely sympatric. Strong support for separate taxa would be found when all lines of evidence show some level of differentiation. However, our examples in the genus *Casearia* showed that often only one criterium applies, whereas the others do not exhibit divergent patterns. Even so, our application of an integrative taxonomy approach by scrutinizing various lines of evidence (Yeates et al., 2011; Mayo, 2022) provided the basis for an informed of the taxa in question. The variable combination of the three lines of evidence used here (morphology, ecology, and distribution), based on the underlying phylogeny, can be summarized in a decision matrix (Figure 6).

### 4.3 Placing our results in the context of previous taxonomic treatments

*Casearia grandiflora,* although retrieved as a nested singleton within *C. arborea,* presented some degree of phenotypical differentiation, whereas ecological and chorological data remained inconclusive. We therefore conclude that *C. grandiflora* should be maintained as a separate species based on at least one line of evidence (Table 4). According to Sleumer (1980), the two species are hard to distinguish when sterile, but some flower characters such as the presence of a peduncle in *C. arborea* versus sessile flowers in *C. grandiflora* allow a clear differentiation of fertile material, thus supporting the distinction of the two species.

**TABLE 4:** Results of analyses and possible outcome on species delimitation

	Phenotype	Ecological niche	Distribution	Taxon concept
*C. arborea* & *C. grandiflora*	Differentiated	similar but not equivalent, not differentiated, high niche overlap	Sympatric, *C. arborea* with a broader range	Different species
*C. arborea* & *C. manausensis*	Differentiated	similar but not equivalent, nested, partial niche overlap	*C. grandiflora* nested within *C. arborea*	(1) Maybe different species or (2) Phenotypically differentiated ecotypes
*C. mariquitensis* & *C. mollis*	Differentiated	similar and equivalent, not differentiated high niche overlap	Sympatric, *C. mollis* also present in Cuba	(1) Different species or (2) Infraspecific divergence
*C. sylvestris* & *C. selloana*	Differentiated	similar but not equivalent, not differentiated, high niche overlap	Sympatric, *C. sylvestris* with a broader range	(1) Different species or (2) Infraspecific divergence
*C. sylvestris* & *C. zizyphoides*	Differentiated	similar but not equivalent, nested, high niche overlap	Sympatric, *C. sylvestris* with a broader range	(1) Different species or (2) Infraspecific divergence

*Casearia manausensis* shows strong phenotypical differentiation toward *C. arborea*, but is narrowly nested within the environmental niche space of the latter. Compared to the wide distribution of *C. arborea* throughout the Neotropics, from Central America to Southern Brazil and into the Caribbean, *C. manausensis* is restricted to a small area within the Amazon. This points to a speciation process within an area subset, where a widely distributed species gave rise to a species with a much smaller range, by ecological differentiation (Rundle & Nosil, 2005; Foote, 2018) and perhaps parapatric speciation.

A sequenced singleton of *Casearia mariquitensis* from Guyana appeared among the samples of *C. mollis*. While there is some morphological differentiation, the ecological analysis revealed considerable overlap. Olson et al. (1999) stated that *C. mariquitensis* and *C. mollis*, along with three other species, *C. decandra* Jacq., *C. arguta* H.B.K. and *C. pitumba*, formed a poorly understood complex. *Casearia mariquitensis* and *C. mollis* were both described in the same work by Kunth (Humboldt et al., 1815), the first based on a type specimen from Colombia, Tolima, and the second with a type from Venezuela (Aragua). Kunth distinguished *C. mariquitensis* as having leaves with an acute base, denticulate margins and being glabrous, whereas *C. mollis* was said to have leaves with a rounded base, dentate margins, and being tomentose beneath. Our morphological analysis largely supported this distinction, although the exact point of delimitation between the two taxa remains unclear.

For *Casearia selloana*, the morphological analysis showed a strong differentiation toward *C. sylvestris* in the PCA, supporting their current treatment as different species. The environmental analysis showed a little ecological differentiation, although the niches of the two species were not fully equivalent. Sleumer (1980) suggested that *C. selloana* might be a variant of *C. sylvestris* in very dry habitats, a notion that remains conflictive given that *C. selloana* is limited to northeastern Brazil, not exactly a dry ecosystem. Notably, *C. sylvestris*, found throughout the New World tropics, encompasses a subspecies *C. sylvestris* subsp. *myricoides* (Griseb.) J.E. Gut., endemic to serpentine areas in Cuba (Gutiérrez, 2000), which is also morphologically distinct by having smaller leaves. This particular case of adaptation to soil type (Borhidi, 1991; Reeves et al., 1999) was not investigated for the five cases analyzed here but should be considered for future assessments. *Casearia sylvestris* shows considerable phylogenetic structure already based on a few loci, which suggests that it could represent a species complex, and so the nested position of *C. selloana* and especially *C. zizyphoides* may eventually be resolved as reciprocally monophyletic.

### 4.4 Handling singletons in the context of an integrative taxonomy approach

Assessing taxon validity by analyzing only line of evidence is not enough and can result in an over‐ or underestimation of species numbers (Carstens et al., 2013). Therefore, studying multiple lines of evidence is crucial as it allows to take into account the various mechanisms involved in speciation (Schlick‐Steiner et al., 2010). Whereas morphological evidence has been very frequently matched with phylogenetic or phylogeographic trees and networks to illuminate species limits and support species classification (Huang et al., 2016; Šmíd et al., 2017; Perkins, 2019; Yang et al., 2021; Andriamihaja et al., 2022), the inclusion of ecological data to achieve this goal is more recent (Boucher et al., 2016; Duan et al., 2019). Recently, various studies have successfully focused on solving species limits with the combined use of molecular analysis, morphometric with PCA or multidimensional scaling analysis (MSA), and ecological niche modeling or niche equivalency or niche similarity tests (Prata et al., 2018; Frajman et al., 2019; Lin et al., 2021). Such investigations typically employ a dedicated study design, including field collection of representative material, to ensure that all specimens can be consistently examined for all kinds of data. In the present case, we adopted this approach to assess the status of nested singletons in phylogenetic analysis. The main difference toward the above‐mentioned studies is that we do not have a congruent sample for both molecular and nonmolecular data. In contrast, given that the molecular sample is by definition a single specimen, hence without any statistic power, we use the analysis of a broad sample of nonsequenced specimens as a proxy to assess the potential status of the taxon represented by the nested singleton. In doing so, we provide a quantitative framework using three lines of evidence (morphology, ecology, and distribution) to interpret the status of a taxon beyond the limited and inconclusive molecular information. Given that nested singletons are a frequent problem in published molecular phylogenies, and given that their status us usually not assessed, thus leaving unresolved taxonomies, our approach appears to be a useful strategy to bridge the lack of more abundant molecular data for the taxa in question.

In this investigation, we want to explore the use of morphological, ecological, and distributional data (Table 4) for delimitating species when taxon sampling in the available molecular trees is limited and molecular phylogenetic analyses alone remain inconclusive to support taxonomic treatments at species level. Specifically, we addressed singletons found in our phylogenetic analysis of *Casearia* as an exemplar.

Our approach shows that quantitative evaluation of nonsequenced specimens that were identified based on morphological characters and using existing prephylogenetic treatments can be successful in evaluating the status of so‐called nested singletons that were found in phylogenetic analyses. Such singletons are frequent in published molecular phylogenetic trees based on multiple sequence alignments of few to multiple loci (Bengtson et al., 2021; Lu‐Irving et al., 2021; García‐Moro et al., 2022) and as well in phylogenomic analyses using RAD (Böhnert et al., 2022) or hyb seq data (Jones et al., 2019; Xu & Chen, 2021). Under normal circumstances, one would target several specimens of a species complex to address species delimitation, then also ideally combining molecular, morphological, ecological, and distributional data in an integrative taxonomy approach. However, singletons are usually the result of nontargeted sampling, i.e., such taxa have not been specifically targeted and they are included as singletons in phylogenetic analysis either because the overarching question is at a different taxonomic level (e.g. genus delimitation or genus placement) or because they represent opportunistic sampling within a larger clade. Still, the respective phylogenetic trees provide useful information for species delimitation and challenge currently used taxon concepts at species level. In such cases, our strategy offers an effective approach: initial hypothesis of potential synonymy due to nested phylogenetic placement, subsequent testing using quantitative morphology, ecology, and distribution of numerous nonsequenced samples taxonomically identified as a given species. These results will make taxon hypotheses explicit, also with respect to data deficiencies and inform targeted sampling in future studies. Our approach will be relevant to assess the status of taxa in case further sampling is logistically challenging but taxonomic decisions are needed in a timely manner such as for completing checklists and flora treatments or the evaluation of the conservation status.

Integrative taxonomy has sometimes been considered as a “solution to the plurality of existing species concepts” (Dayrat, 2005; Schlick‐Steiner et al., 2010). Considering that there are different (biological) species concepts that connect to particular speciation mechanisms in flowering plants, we argue that in many cases of hypothesized species, the challenge is to obtain sufficient evidence (both molecular and nonmolecular) to unravel which species concept will precisely apply. Morphological, ecological, and geographical data can provide evidence in favor of speciation hypotheses such as allopatric, parapatric, or petripatric, which by themselves have a spatial dimension. Moreover, they allow to include the wealth of existing specimens in herbaria. We further observe that phylogenomic analyses increase the resolution within shallow clades, encompassing one to several putative species (Prata et al., 2018; Lin et al., 2021; Smith et al., 2022). However, the delimitation of species, and the subsequent circumscription and naming of taxa, from the background of the molecular topology is usually being done by matching morphological character states to parts of the topology, underscoring the relevance of an integrated taxonomic approach. Phylogenomic analyses with a population‐level sampling to represent the genetic diversity within putative species in order to inform model approaches to recognize discontinuities resulting from speciation are still rare due to their complexity and the high effort that they require.

## AUTHOR CONTRIBUTIONS

**Astrid de Mestier:** Conceptualization (equal); investigation (lead); methodology (equal); writing – original draft (lead); writing – review and editing (equal). **Robert Lücking:** Conceptualization (equal); investigation (supporting); methodology (equal); writing – original draft (supporting); writing – review and editing (equal). **Jorge Gutierrez Amaro:** Conceptualization (equal); investigation (supporting); writing – review and editing (supporting). **Marcela Celis:** Conceptualization (equal); investigation (supporting); writing – review and editing (supporting). **Grischa Brokamp:** Conceptualization (equal); investigation (supporting); writing – review and editing (supporting). **Thomas Borsch:** Conceptualization (equal); investigation (supporting); methodology (equal); writing – original draft (supporting); writing – review and editing (equal).

## CONFLICT OF INTEREST

The authors declare that they have no conflict of interests.

## DATA AVAIBILITY STATEMENT

The sequences used in this study are available in NCBI https://www.ncbi.nlm.nih.gov.

## Supporting information


Appendix S1.

Appendix S2.

Appendix S3.

Appendix S4.

Appendix S5.

Table S1.

Table S2.

Table S3.

## References

[ece39736-bib-0094] Šmíd, J. , Kalousová, M. , Mandák, B. , Houška, J. , Chládová, A. , Pinedo, M. , & Lojka, B. (2017). Morphological and genetic diversity of camu‐camu [Myrciaria dubia (Kunth) McVaugh] in the Peruvian Amazon. PLoS One, 12, e0179886. 10.1371/JOURNAL.PONE.0179886 28658316 PMC5489195

[ece39736-bib-0001] Agapow, P.‐M. , Bininda‐Emonds, O. R. P. , Crandall, K. A. , Gittleman, J. L. , Mace, G. M. , Marshall, J. C. , & Purvis, A. (2004). The impact of species concept on biodiversity studies. The Quarterly Review of Biology, 79, 161–179. 10.1086/383542 15232950

[ece39736-bib-0002] Alford, M. H. (2005). Systematic studies in Flacourtiaceae.

[ece39736-bib-0003] Andriamihaja, C. F. , Botomanga, A. , Misandeau, C. , Ramarosandratana, A. V. , Grisoni, M. , Da Silva, D. , Pailler, T. , Jeannoda, V. H. , & Besse, P. (2022). Integrative taxonomy and phylogeny of leafless *vanilla* orchids from the south‐West Indian Ocean region reveal two new Malagasy species. Journal of Systematics and Evolution. 10.1111/JSE.12858

[ece39736-bib-0004] Applequist, W. L. , & Gates, M. T. (2020). Two new small‐leaved species of Casearia (Salicaceae) from Madagascar. Novon, A Journal for Botanical Nomenclature, 28, 256–262. 10.3417/2020611

[ece39736-bib-0006] Böhnert, T. , Luebert, F. , Merklinger, F. F. , Harpke, D. , Stoll, A. , Schneider, J. V. , Blattner, F. R. , Quandt, D. , & Weigend, M. (2022). Plant migration under long‐lasting hyperaridity – Phylogenomics unravels recent biogeographic history in one of the oldest deserts on earth. The New Phytologist, 234, 1863–1875. 10.1111/NPH.18082 35274308

[ece39736-bib-0026] DRYFLOR , Banda, K. R. , Delgado‐Salinas, A. , Dexter, K. G. , Linares‐Palomino, R. , Oliveira‐Filho, A. , Prado, D. , Pullan, M. , Quintana, C. , Riina, R. , Rodríguez, G. M. , Weintritt, J. , Acevedo‐Rodríguez, P. , Adarve, J. , Álvarez, E. , Anairamiz Aranguren, B. , Arteaga, J. C. , Aymard, G. , Castaño, A. , … Pennington, R. T. (2016). Plant diversity patterns in neotropical dry forests and their conservation implications. Science, 353, 1383–1387. 10.1126/SCIENCE.AAF5080 27708031

[ece39736-bib-0005] Bengtson, A. , Osborne, J. , & Anderberg, A. A. (2021). Phylogeny of Anisopappus with species circumscriptions revisited (Asteraceae: Athroismeae). Taxon, 70, 351–364. 10.1002/TAX.12448

[ece39736-bib-0007] Borhidi, A. (1991). Phytogeography and vegetation ecology of Cuba. Akaémiai K.

[ece39736-bib-0008] Borsch, T. , Berendsohn, W. , Dalcin, E. , Delmas, M. , Demissew, S. , Elliott, A. , Fritsch, P. , Fuchs, A. , Geltman, D. , Güner, A. , Haevermans, T. , Knapp, S. , Roux, M. M. , Loizeau, P. A. , Miller, C. , Miller, J. , Miller, J. T. , Palese, R. , Paton, A. , … Zamora, N. (2020). World Flora online: Placing taxonomists at the heart of a definitive and comprehensive global resource on the world's plants. Taxon, 69, 1311–1341. 10.1002/tax.12373

[ece39736-bib-0009] Borsch, T. , Flores‐Olvera, H. , Zumaya, S. , & Müller, K. (2018). Pollen characters and DNA sequence data converge on a monophyletic genus *Iresine* (Amaranthaceae, Caryophyllales) and help to elucidate its species diversity. Taxon, 67, 944–976. 10.12705/675.7

[ece39736-bib-0010] Borsch, T. , Hernández‐Ledesma, P. , Berendsohn, W. , Flores‐Olvera, H. , Ochoterena, H. , Zuloaga, F. O. , et al. (2015). An integrative and dynamic approach for monographing species‐rich plant groups – Building the global synthesis of the angiosperm order Caryophyllales. Perspectives in Plant Ecology, Evolution and Systematics, 17, 284–300. 10.1016/j.ppees.2015.05.003

[ece39736-bib-0011] Boucher, F. C. , Zimmermann, N. E. , & Conti, E. (2016). Allopatric speciation with little niche divergence is common among alpine Primulaceae. Journal of Biogeography, 43, 591–602. 10.1111/JBI.12652

[ece39736-bib-0012] Breteler, F. J. (2008). A synopsis of Casearia Jacq. (Samydeae‐Salicaceae) in west and Central Africa with a description of a new species from eastern Congo (Kinshasa). Kew Bulletin, 63, 101–112. 10.1007/s12225-007-9005-7

[ece39736-bib-0013] Bromham, L. , Woolfit, M. , Lee, M. S. Y. , & Rambaut, A. (2002). Testing the relationship between morphological and molecular rates of change along phylogenies. Evolution, 56, 1921–1930. 10.1111/J.0014-3820.2002.TB00118.X 12449479

[ece39736-bib-0014] Carnicero, P. , Schönswetter, P. , Garcia‐Jacas, N. , & Galbany‐Casals, M. (2019). Is there a need for accepting paraphyletic taxa? A case study in the Sardinian endemic *Cymbalaria muelleri* (Plantaginaceae). Botanical Journal of the Linnean Society, 191, 325–338. 10.1093/botlinnean/boz052

[ece39736-bib-0015] Carstens, B. C. , Pelletier, T. A. , Reid, N. M. , & Satler, J. D. (2013). How to fail at species delimitation. Molecular Ecology, 22, 4369–4383. 10.1111/mec.12413 23855767

[ece39736-bib-0016] Charrad, M. , Ghazzali, N. , Boiteau, V. , & Niknafs, A. (2014). NbClust: An r package for determining the relevant number of clusters in a data set. Journal of Statistical Software, 61, 1–36.

[ece39736-bib-0017] Comes, H. P. (2004). The Mediterranean region – A hotspot for plant biogeographic research. The New Phytologist, 164, 11–14. 33873489 10.1111/j.1469-8137.2004.01194.x

[ece39736-bib-0018] Correll, D. S. , & Correll, H. B. (1982). The flora of the Bahama archipelago. Cramer. Vaduz, Liechtenstein.

[ece39736-bib-0019] Crisp, M. D. , & Chandler, G. T. (1996). Paraphyletic species. Telopea, 6, 813–844. 10.7751/telopea19963037

[ece39736-bib-0020] DANE . (2017). Capa de referencia de verreda Vigencia.

[ece39736-bib-0021] Darriba, D. , Taboada, G. L. , Doallo, R. , & Posada, D. (2012). JModelTest 2: More models, new heuristics and parallel computing. Nature Methods, 9, 772. 10.1038/nmeth.2109 PMC4594756 22847109

[ece39736-bib-0022] Dayrat, B. (2005). Towards integrative taxonomy. Biological Journal of the Linnean Society, 85, 407–415. 10.1111/j.1095-8312.2005.00503.x

[ece39736-bib-0023] De Smedt, S. , Bogaerts, A. , Groom, Q. , & Engledow, H. (2018). Botanicalcollections.be: The new virtual herbarium of Meise botanic garden (BR). Biodiversity Information Science and Standards, 2, e26140. 10.3897/BISS.2.26140

[ece39736-bib-0024] Devecchi, M. F. , Lovo, J. , Moro, M. F. , Andrino, C. O. , Barbosa‐Silva, R. G. , Viana, P. L. , Giulietti, A. M. , Antar, G. , Watanabe, M. T. C. , & Zappi, D. C. (2020). Beyond forests in the Amazon: Biogeography and floristic relationships of the Amazonian savannas. Botanical Journal of the Linnean Society, 193, 478–503. 10.1093/botlinnean/boaa025

[ece39736-bib-0025] Di Cola, V. , Broennimann, O. , Petitpierre, B. , Breiner, F. T. , D'Amen, M. , Randin, C. , Engler, R. , Pottier, J. , Pio, D. , Dubuis, A. , Pellissier, L. , Mateo, R. G. , Hordijk, W. , Salamin, N. , & Guisan, A. (2017). Ecospat: An R package to support spatial analyses and modeling of species niches and distributions. Ecography, 40, 774–787. 10.1111/ecog.02671

[ece39736-bib-0027] Duan, L. , Harris, A. J. , Ye, W. , Deng, S.‐W. , Song, Z.‐Q. , Chen, H.‐F. , & Wen, J. (2019). Untangling the taxonomy of the Cladrastis clade (Leguminosae: Papilionoideae) by integrating phylogenetics and ecological evidence. Taxon, 68, 1189–1203. 10.1002/tax.12155

[ece39736-bib-0028] Edwards, D. L. , & Knowles, L. L. (2014). Species detection and individual assignment in species delimitation: Can integrative data increase efficacy? Proceedings of the Royal Society B: Biological Sciences, 281, 20132765. 10.1098/RSPB.2013.2765 PMC3896021 24403337

[ece39736-bib-0029] Fišer, C. , Robinson, C. T. , & Malard, F. (2018). Cryptic species as a window into the paradigm shift of the species concept. Molecular Ecology, 27, 613–635. 10.1111/mec.14486 29334414

[ece39736-bib-0030] Foote, A. D. (2018). Sympatric speciation in the genomic era. Trends in Ecology & Evolution, 33, 85–95. 10.1016/J.TREE.2017.11.003 29198471

[ece39736-bib-0031] Frajman, B. , Záveská, E. , Gamisch, A. , Moser, T. , Arthofer, W. , Hilpold, A. , et al. (2019). Integrating phylogenomics, phylogenetics, morphometrics, relative genome size and ecological niche modelling disentangles the diversification of Eurasian euphorbia seguieriana s. l. (Euphorbiaceae). Molecular Phylogenetics and Evolution, 134, 238–252. 10.1016/J.YMPEV.2018.10.046 30415023

[ece39736-bib-0032] Frost, L. A. , O'Leary, N. , Lagomarsino, L. P. , Tank, D. C. , & Olmstead, R. G. (2021). Phylogeny, classification, and character evolution of tribe Citharexyleae (Verbenaceae). American Journal of Botany, 108, 1982–2001. 10.1002/AJB2.1750 34669193

[ece39736-bib-0033] García‐Moro, P. , Otero, A. , Benítez‐Benítez, C. , Costa, L. , Martín‐Bravo, S. , Naczi, R. F. C. , Reznicek, A. A. , Roalson, E. H. , Starr, J. R. , & Jiménez‐Mejías, P. (2022). Biogeography and systematics of Carex subgenus Uncinia (Cyperaceae): A unique radiation for the genus Carex in the southern hemisphere. Taxon, 71, 587–607. 10.1002/TAX.12678

[ece39736-bib-0034] GoogleInc . (2020). Google Earth, version 7.3.3.7786.

[ece39736-bib-0035] Guenser, P. , Ginot, S. , Escarguel, G. , & Goudemand, N. (2022). When less is more and more is less: The impact of sampling effort on species delineation. Palaeontology, 65, e12598. 10.1111/PALA.12598

[ece39736-bib-0036] Gutiérrez, J. (2000). Flacourtiaceae 5(1). In Flora de la Republica de Cuba. Koeltz Scientific Books.

[ece39736-bib-0041] Hörandl, E. , & Stuessy, T. F. (2010). Paraphyletic groups as natural units of biological classification. Taxon, 59, 1641–1653. 10.1002/tax.596001

[ece39736-bib-0037] Hennig, W. (1950). Grundzüge einer Theorie der phylogenetischen Systematik. Deutscher Zentralverlag.

[ece39736-bib-0038] Hennig, W. (1966). Phylogenetic systematics. University of Illinois Press.

[ece39736-bib-0039] Hillis, D. M. (1987). Molecular versus morphological approaches to systematics. Annual Review of Ecology, Evolution, and Systematics, 18, 23–42.

[ece39736-bib-0040] Hinchliff, C. E. , Smith, S. A. , Allman, J. F. , Burleigh, J. G. , Chaudhary, R. , Coghill, L. M. , Crandall, K. A. , Deng, J. , Drew, B. T. , Gazis, R. , Gude, K. , Hibbett, D. S. , Katz, L. A. , Laughinghouse, H. D., IV , McTavish, E. J. , Midford, P. E. , Owen, C. L. , Ree, R. H. , Rees, J. A. , … Cranston, K. A. (2015). Synthesis of phylogeny and taxonomy into a comprehensive tree of life. Proceedings of the National Academy of Sciences of the United States of America, 112, 12764–12769. 10.5061/dryad.8j60q 26385966 PMC4611642

[ece39736-bib-0042] Howard, R. (1989). Flora of the lesser Antilles 6(3). Arnold Arboretum, Harvard University.

[ece39736-bib-0043] Huang, C. H. , Sun, R. , Hu, Y. , Zeng, L. , Zhang, N. , Cai, L. , Zhang, Q. , Koch, M. A. , al‐Shehbaz, I. , Edger, P. P. , Pires, J. C. , Tan, D. Y. , Zhong, Y. , & Ma, H. (2016). Resolution of Brassicaceae phylogeny using nuclear genes uncovers nested radiations and supports convergent morphological evolution. Molecular Biology and Evolution, 33, 394–412. 10.1093/MOLBEV/MSV226 26516094 PMC4866547

[ece39736-bib-0044] Humboldt, A. , Bonpland, A. , & Kunth, C. S. (1815). Nova genera et species plantarum (quarto ed.). Librairie Grecque, Latine, Allemande.

[ece39736-bib-0045] James, S. A. , Soltis, P. S. , Belbin, L. , Chapman, A. D. , Nelson, G. , Paul, D. L. , & Collins, M. (2018). Herbarium data: Global biodiversity and societal botanical needs for novel research: Global. Applications in Plant Sciences, 6, e1024. 10.1002/aps3.1024 29732255 PMC5851569

[ece39736-bib-0046] Jones, K. E. , Fér, T. , Schmickl, R. E. , Dikow, R. B. , Funk, V. A. , Herrando‐Moraira, S. , Johnston, P. R. , Kilian, N. , Siniscalchi, C. M. , Susanna, A. , Slovák, M. , Thapa, R. , Watson, L. E. , & Mandel, J. R. (2019). An empirical assessment of a single family‐wide hybrid capture locus set at multiple evolutionary timescales in Asteraceae. Applications in Plant Sciences, 7, e11295. 10.1002/APS3.11295 31667023 PMC6814182

[ece39736-bib-0111] Kaplan . (2020). fastdummies. Fast creation of dummy (binary) columns and rows from categorical variables. R package version 1.6.0.

[ece39736-bib-0047] Kato, M. , Werukamkul, P. , Won, H. , & Koi, S. (2019). Paraphyletic species of Podostemaceae: *Cladopus fallax* and *Polypleurum wallichii* . Phytotaxa, 401, 33–48. 10.11646/phytotaxa.401.1.3

[ece39736-bib-0048] Kilian, N. , Henning, T. , Plitzner, P. , Müller, A. , Güntsch, A. , Stöver, B. C. , Müller, K. F. , Berendsohn, W. G. , & Borsch, T. (2015). Sample data processing in an additive and reproducible taxonomic workflow by using character data persistently linked to preserved individual specimens. Database, 2015, 94. 10.1093/DATABASE/BAV094 PMC4589695 26424081

[ece39736-bib-0049] Klazenga, N. (2018). The Australasian virtual herbarium (AVH) and the changing role of herbaria. Biodiversity Information Science and Standards, 2, e25866. 10.3897/BISS.2.25866

[ece39736-bib-0110] Löhne, C. , & Borsch, T. (2005). Molecular evolution and phylogenetic utility of the petD group II intron: A case study in basal angiosperms. Molecular Biology and Evolution, 22(2), 317–332. 10.1093/molbev/msi019 15496557

[ece39736-bib-0050] Le Bras, G. , Pignal, M. , Jeanson, M. L. , Muller, S. , Aupic, C. , Carré, B. , Flament, G. , Gaudeul, M. , Gonçalves, C. , Invernón, V. R. , Jabbour, F. , Lerat, E. , Lowry, P. P. , Offroy, B. , Pimparé, E. P. , Poncy, O. , Rouhan, G. , & Haevermans, T. (2017). The French Muséum national d'histoire naturelle vascular plant herbarium collection dataset. Scientific Data, 4, 1–16. 10.1038/sdata.2017.16 PMC5308200 28195585

[ece39736-bib-0051] Leroy, B. , Meynard, C. N. , Bellard, C. , & Courchamp, F. (2016). Virtualspecies, an R package to generate virtual species distributions. Ecography, 39, 599–607. 10.1111/ecog.01388

[ece39736-bib-0052] Lexer, C. , & Widmer, A. (2008). Review. The genic view of plant speciation: Recent progress and emerging questions. Philosophical Transactions of the Royal Society of London. Series B, Biological Sciences, 363, 3023–3036. 10.1098/rstb.2008.0078 18579476 PMC2607315

[ece39736-bib-0053] Lin, H. Y. , Gu, K. J. , Li, W. H. , & Zhao, Y. P. (2021). Integrating coalescent‐based species delimitation with ecological niche modeling delimited two species within the Stewartia sinensis complex (Theaceae). Journal of Systematics and Evolution, 60(5), 1037–1048. 10.1111/JSE.12732/SUPPINFO

[ece39736-bib-0054] Linnaeus, C. (1753). Species plantarum, exhibentes plantas rite cognitas, ad genera relatas, cun differentiis specificis, nominibus tribialibus, synonymis selectis, locis natalibus, secundum systema sexuale digestas. Laurentii Salvi.

[ece39736-bib-0055] Liogier, H. A. (1994). Descriptive flora of Puerto Rico and adjacent islands, 3. Editorial de la Universidad de Puerto Rico.

[ece39736-bib-0056] Lu‐Irving, P. , Bedoya, A. M. , Salimena, F. R. G. , dos Santos Silva, T. R. , Viccini, L. F. , Bitencourt, C. , Thode, V. A. , Cardoso, P. H. , O'Leary, N. , & Olmstead, R. G. (2021). Phylogeny of lantana, Lippia, and related genera (Lantaneae: Verbenaceae). American Journal of Botany, 108, 1354–1373. 10.1002/AJB2.1708 34418063

[ece39736-bib-0065] Müller, J. , Müller, K. , Neinhuis, C. , & Quandt, D. (2010). PhyDe: Phylogenetic data editor, Version 0.9971.

[ece39736-bib-0066] Müller, K. (2004). PRAP—Computation of Bremer support for large data sets. Molecular Phylogenetics and Evolution, 31, 780–182. 15062810 10.1016/j.ympev.2003.12.006

[ece39736-bib-0057] Majure, L. C. , Barrios, D. , Díaz, E. , Zumwalde, B. A. , Testo, W. , & Negrón‐Ortíz, V. (2021). Pleistocene aridification underlies the evolutionary history of the Caribbean endemic, insular, giant Consolea (Opuntioideae). American Journal of Botany, 108, 200–215. 10.1002/AJB2.1610 33598914

[ece39736-bib-0058] Mansion, G. , Parolly, G. , Crowl, A. A. , Mavrodiev, E. , Cellinese, N. , Oganesian, M. , Fraunhofer, K. , Kamari, G. , Phitos, D. , Haberle, R. , Akaydin, G. , Ikinci, N. , Raus, T. , & Borsch, T. (2012). How to handle speciose clades? Mass taxon‐sampling as a strategy towards illuminating the natural history of campanula (Campanuloideae). PLoS One, 7, e50076. 10.1371/JOURNAL.PONE.0050076 23209646 PMC3509159

[ece39736-bib-0059] Marquete, R. , & Mansano, V. F. (2010). A new species of *Casearia* (Salicaceae) from southeastern Brazil. Novon, A Journal for Botanical Nomenclature, 20, 179–181. 10.3417/2009011

[ece39736-bib-0060] Marquete, R. , & Mansano, V. F. (2012). Taxonomic revision of the *Casearia ulmifolia* Complex (Salicaceae). Novon, A Journal for Botanical Nomenclature, 22, 196–206. 10.3417/2011011

[ece39736-bib-0061] Mayo, S. J. (2022). Plant taxonomic species and their role in the workflow of integrative species delimitation. Kew Bulletin, 77, 1–26. 10.1007/S12225-022-10002-X

[ece39736-bib-0062] Mayo, S. J. , Allkin, R. , Baker, W. , Blagoderov, V. , Brake, I. , Clark, B. , Govaerts, R. , Godfray, C. , Haigh, A. , Hand, R. , Harman, K. , Jackson, M. , Kilian, N. , Kirkup, D. W. , Kitching, I. , Knapp, S. , Lewis, G. P. , Malcolm, P. , von Raab‐Straube, E. , … Wilkin, P. (2008). Alpha e‐taxonomy: Responses from the systematics community to the biodiversity crisis. Kew Bulletin, 63, 1–16. 10.1007/S12225-008-9014-1

[ece39736-bib-0063] Mestier, A. d. , Brokamp, G. , Celis, M. , Falcón‐Hidalgo, B. , Gutiérrez, J. , & Borsch, T. (2022). Character evolution and biogeography of Casearia (Salicaceae): Evidence for the south American origin of a pantropical genus and for multiple migrations to the Caribbean islands. Taxon, 71, 321–347. 10.1002/TAX.12656

[ece39736-bib-0064] Miller, M. A. , Pfeiffer, W. , & Schwartz, T. (2011). The CIPRES science gateway: A community resource for phylogenetic analyses. in *Proceedings of the TeraGrid 2011 Conference: Extreme digital discovery, TG’11* (New York, New York, USA: ACM press), 1. 10.1145/2016741.2016785

[ece39736-bib-0067] Naciri, Y. , & Linder, H. P. (2015). Species delimitation and relationships: The dance of the seven veils. Taxon, 64, 3–16. 10.12705/641.24

[ece39736-bib-0068] Nepomuceno, Á. , & Alves, M. (2020). Salicaceae in the northern portion of the Atlantic Forest. Rodriguesia, 71, e01232018.2020. 10.1590/2175-7860202071133

[ece39736-bib-0069] Nixon, K. C. (1999). The parsimony ratchet, a new method for rapid parsimony analysis. Cladistics, 15, 407–414. 10.1006/clad.1999.0121 34902938

[ece39736-bib-0070] Olson, M. , Berry, P. E. , & Aymard, C. (1999). Flacourtiaceae. In Flora of the Venezuelan Guayana (Vol. 5, pp. 434–474). Missouri Botanical Garden Press.

[ece39736-bib-0071] Padial, J. M. , Miralles, A. , De la Riva, I. , & Vences, M. (2010). The integrative future of taxonomy. Frontiers in Zoology, 7, 16. 10.1186/1742-9994-7-16 20500846 PMC2890416

[ece39736-bib-0072] Pante, E. , Schoelinck, C. , & Puillandre, N. (2015). From integrative taxonomy to species description: One step beyond. Systematic Biology, 64, 152–160. 10.1093/sysbio/syu083 25358968

[ece39736-bib-0073] Perkins, A. J. (2019). Molecular phylogenetics and species delimitation in annual species of Hydrocotyle (Araliaceae) from South Western Australia. Molecular Phylogenetics and Evolution, 134, 129–141. 10.1016/j.ympev.2019.02.011 30771512

[ece39736-bib-0074] Prata, E. M. B. , Sass, C. , Rodrigues, D. P. , Domingos, F. M. C. B. , Specht, C. D. , Damasco, G. , Ribas, C. C. , Fine, P. V. A. , & Vicentini, A. (2018). Towards integrative taxonomy in neotropical botany: Disentangling the *Pagamea guianensis* species complex (Rubiaceae). Botanical Journal of the Linnean Society, 188, 213–231. 10.1093/BOTLINNEAN/BOY051

[ece39736-bib-0075] QGIS association . (2020). QGIS Geographic Information System. Available at: http://www.qgis.org

[ece39736-bib-0083] Rønsted, N. , Grace, O. M. , & Carine, M. A. (2020). Editorial: Integrative and translational uses of herbarium collections across time, space, and species. Frontiers in Plant Science, 11, 1319. 10.3389/fpls.2020.01319 32973855 PMC7472523

[ece39736-bib-0076] Rambaut, A. (2010). FigTree, v1.4.4. Available at: http://tree.bio.ed.ac.uk/software/figtree/

[ece39736-bib-0077] Ravinet, M. , Faria, R. , Butlin, R. K. , Galindo, J. , Bierne, N. , Rafajlović, M. , Noor, M. A. F. , Mehlig, B. , & Westram, A. M. (2017). Interpreting the genomic landscape of speciation: A road map for finding barriers to gene flow. Journal of Evolutionary Biology, 30, 1450–1477. 10.1111/jeb.13047 28786193

[ece39736-bib-0078] RCoreTeam . (2013). A language and environment for statistical computing. R Found. Stat. Comput Available at: http://www.r‐project.org/

[ece39736-bib-0079] Reeves, R. D. , Baker, A. J. M. , Borhidi, A. , & Berazaín, R. (1999). Nickel hyperaccumulation in the serpentine Flora of Cuba. Annals of Botany, 83, 29–38. 10.1006/ANBO.1998.0786

[ece39736-bib-0080] Robertson, T. , Döring, M. , Guralnick, R. , Bloom, D. , Wieczorek, J. , Braak, K. , Otegui, J. , Russell, L. , & Desmet, P. (2014). The GBIF integrated publishing toolkit: Facilitating the efficient publishing of biodiversity data on the internet. PLoS One, 9, e102623. 10.1371/journal.pone.0102623 25099149 PMC4123864

[ece39736-bib-0081] Rojas‐Soto, O. R. , Navarro‐Siguenza, A. G. , & Espinosa de los Monteros, A. (2010). Systematics and bird conservation policies: The importance of species limits. Bird Conservation International, 20, 176–185. 10.1017/S0959270909990268

[ece39736-bib-0082] Ronquist, F. , Huelsenbeck, J. P. , & Teslenko, M. (2011). Mr Bayes version 3.2 manual: Tutorials and models summaries. Available at: http://mrbayes.sourceforge.net/mb3.2_manual.Pdf

[ece39736-bib-0084] Ruiz‐Sanchez, E. , & Londoño, X. (2017). *Otatea colombiana* (Poaceae: Bambusoideae: Bambuseae: Guaduinae), a new species endemic to Colombia. Systematic Botany, 42, 817–822. 10.1600/036364417X696492

[ece39736-bib-0085] Rundle, H. D. , & Nosil, P. (2005). Ecological speciation. Ecology Letters, 8, 336–352. 10.1111/j.1461-0248.2004.00715.x

[ece39736-bib-0086] Scherz, M. D. , Vences, M. , Rakotoarison, A. , & Andreone, F. (2017). Lumping or splitting in the Cophylinae (Anura: Microhylidae) and the need for a parsimony of taxonomic changes: A response to Peloso et al. (2017). Salamandra, 53, 479–483 Available at: http://www.salamandra‐journal.com [Accessed May 30, 2022]

[ece39736-bib-0087] Schlick‐Steiner, B. C. , Steiner, F. M. , Seifert, B. , Stauffer, C. , Christian, E. , & Crozier, R. H. (2010). Integrative taxonomy: A multisource approach to exploring biodiversity. Annual Review of Entomology, 55, 421–438. 10.1146/annurev-ento-112408-085432 19737081

[ece39736-bib-0088] Schneider, C. A. , Rasband, W. S. , & Eliceiri, K. W. (2012). NIH image to ImageJ: 25 years of image analysis. Nature Methods, 9, 671–675. 10.1038/nmeth.2089 22930834 PMC5554542

[ece39736-bib-0089] Seregin, A. P. (2018). The largest digital herbarium in Russia is now available online! Taxon, 67, 465–467.

[ece39736-bib-0090] Sheridan, J. A. , & Stuart, B. L. (2018). Hidden species diversity in Sylvirana nigrovittata (amphibia: Ranidae) highlights the importance of taxonomic revisions in biodiversity conservation. PLoS One, 13, e0192766. 10.1371/JOURNAL.PONE.0192766 29538432 PMC5851555

[ece39736-bib-0091] Simmons, M. P. , & Ochoterena, H. (2000). Gaps as characters in sequence‐based phylogenetic analyses. Systematic Biology, 49, 369–381. 12118412

[ece39736-bib-0092] Simpson, G. G. (1951). The species concept. Evolution, 5, 285. 10.2307/2405675

[ece39736-bib-0093] Sleumer, H. O. (1980). Flacourtiaceae. In Flora Neotropica (pp. 1–499). New York Botanical Garden Press.

[ece39736-bib-0095] Smith, L. T. , Magdalena, C. , Przelomska, N. A. S. , Perez‐Escobar, O. , Melgar‐Gomez, D. G. , Beck, S. , et al. (2022). Revised species delimitation in the giant water lily genus Victoria (Nymphaeaceae) confirms a new species and has implications for its conservation. Frontiers in Plant Science, 13, 883151. 10.3389/fpls.2022.883151 35860537 PMC9289450

[ece39736-bib-0097] Stöver, B. C. , & Müller, K. (2010). TreeGraph2: Combining and visualizing evidence from different phylogenetic analyses. BMC Bioinformatics, 11, 7. 10.1186/1471-2105-11-7 20051126 PMC2806359

[ece39736-bib-0096] Stanton, D. W. G. , Frandsen, P. , Waples, R. K. , Heller, R. , Russo, I. R. M. , Orozco‐terWengel, P. A. , Pedersen, C. E. T. , Siegismund, H. R. , & Bruford, M. W. (2019). More grist for the mill? Species delimitation in the genomic era and its implications for conservation. Conservation Genetics, 20, 101–113. 10.1007/s10592-019-01149-5

[ece39736-bib-0098] Stuessy, T. F. (2009). Plant taxonomy: The systematic evaluation of comparative data. Columbia University Press.

[ece39736-bib-0099] Supple, M. A. , & Shapiro, B. (2018). Conservation of biodiversity in the genomics era. Genome Biology, 19, 1–12. 10.1186/S13059-018-1520-3 30205843 PMC6131752

[ece39736-bib-0100] Swofford, D. L. (2008). PAUP: Phylogenetic analysis using parsimony (and other methods). Version 4. Sinauer As. 10.1007/978-1-4020-6754-9_12413

[ece39736-bib-0101] Thiers, B. , Tulig, M. , & Watson, K. (2016). Digitization of the New York botanical garden herbarium. Brittonia, 68, 324–333.

[ece39736-bib-0102] Thompson, J. D. , Lavergne, S. , Affre, L. , Gaudeul, M. , & Debussche, M. (2005). Ecological differentiation of Mediterranean endemic plants. Taxon, 54, 967–976. 10.2307/25065481

[ece39736-bib-0103] Vogel Ely, C. , Bordignon, S. A. L. , Trevisan, R. , & Boldrini, I. I. (2017). Implications of poor taxonomy in conservation. Journal for Nature Conservation, 36, 10–13. 10.1016/J.JNC.2017.01.003

[ece39736-bib-0104] Warburg, O. (1895). Flacourtiaceae. In K. Engler & A. Prantl (Eds.), Die Natürlichen Pflanzenfamilien ed1. vol.3(6) (pp. 1–56). Engelmann.

[ece39736-bib-0105] Warren, D. L. , Richard, E. , & Turelli, M. (2010). ENMTools: A toolbox for comparative studies of environmental niche models. Ecography, 33, 607–611. 10.1111/j.1600-0587.2009.06142.x

[ece39736-bib-0106] Will, K. W. , Mishler, B. D. , & Wheeler, Q. D. (2005). The perils of DNA barcoding and the need for integrative taxonomy. Systematic Biology, 54, 844–851. 10.1080/10635150500354878 16243769

[ece39736-bib-0107] Xu, L. S. , & Chen, Y. S. (2021). Phylogeny, origin, and dispersal of Dubyaea (Asteraceae) based on Hyb‐seq data. Molecular Phylogenetics and Evolution, 164, 107289. 10.1016/J.YMPEV.2021.107289 34371187

[ece39736-bib-0108] Yang, Y. T. , Yang, X. C. , Wang, M. C. , Zhong, L. L. , Ma, R. , Ma, T. , Liu, J. Q. , Davis, C. C. , & Xi, Z. X. (2021). Species delimitation of north American Nyssa species. Journal of Systematics and Evolution, 60, 747–758. 10.1111/JSE.12725

[ece39736-bib-0109] Yeates, D. K. , Seago, A. , Nelson, L. , Cameron, S. L. , Joseph, L. , & Trueman, J. W. H. (2011). Integrative taxonomy, or iterative taxonomy? Systematic Entomology, 36, 209–217. 10.1111/j.1365-3113.2010.00558.x

